# Clinical neuropathology practice guide 5-2012: Updated guideline for the diagnosis of anti-neuronal antibodies 

**DOI:** 10.5414/NP300545

**Published:** 2012-04-18

**Authors:** Romana Höftberger, Josep Dalmau, Francesc Graus

**Affiliations:** 1 Institute of Neurology, Medical University of Vienna, Austria,; 2 Institució Catalana de Recerca i Estudis Avançats (ICREA) - Institut d´Investigacio Biomedica August Pi i Suyer (IDIBAPS), Hospital Clinic, Barcelona, Spain,; 3 Department of Neurology, University of Pennsylvania, Philadelphia, PA, USA,; 4 Service of Neurology, Hospital Clinic-IDIBAPS, Barcelona, Spain

**Keywords:** anti-neuronal antibodies, diagnosis, tissue-based assay, cell-based assay, immunoblot, sensitivity, specificity

## Abstract

In recent years there is an increasing description of novel anti-neuronal antibodies that are associated with paraneoplastic and non-paraneoplastic neurological syndromes. These antibodies are useful in clinical practice to confirm the immunmediated origin of the neurological disorder and are helpful in tumor search. Currently, anti-neuronal antibodies can be classified according to the location of the recognized antigen into two groups, 1.) intraneuronal antigens and 2.) antigens located in the cell membrane. Different techniques are established for detecting these antibodies: tissue-based assay (TBA), cell-based assay (CBA), immunoblot, immunoprecipitation assay (IP), and ELISA. TBA detect most of the antibodies, however, different pretreatment methods of rat brain are necessary to visualize either Group 1 or 2 antibodies. Higher specificity is provided by immunoblots, applicable for Group 1 antibodies, and CBA, suitable for Group 2 antibodies. IP and ELISA may be useful for the detection of specific antibodies or to solve particular issues such as antibody titers. Diagnosis of paraneoplastic and non-paraneoplastic neurological syndromes has important implications on treatment and follow-up of patients. Selection and proper combination of test systems and appropriate knowledge of the clinical information will provide a maximum of sensitivity and specificity in identifying the associated antibody.

## Background 

Neurological syndromes associated with anti-neuronal antibodies are a heterogenous group of autoimmune disorders that can either be linked to an underlying tumor, and are then called paraneoplastic neurological syndromes (PNS), or have an unknown origin as primary autoimmune response against the CNS. The detection of anti-neuronal antibodies influences treatment and follow-up of patients as they confirm the autoimmune-mediated nature of the neurologic syndrome and may be the first indicator for the presence of a tumor. During the last years an increasing number of anti-neuronal antibodies and related syndromes has been described, leading to a continuously changing view on the significance and implications in their diagnostic use [1]. Currently, antibodies are classified into two groups, according to the location of the recognized antigen (Table 1) [2]. Group 1 antibodies are directed against intracellular antigens, therefore they are not considered to be directly involved in the autoimmune attack of neurons. However, they are associated with specific tumors and sometimes identify particular PNS. They are thus useful immunological markers in the diagnosis of the underlying neoplasm. Group 2 antibodies are directed against cell surface antigens, such as synaptic receptors or components of trans-synaptic protein complexes and are supposed to be directly responsible for the neuronal dysfunction. Associated clinical symptoms often comprise different forms of autoimmune encephalitis and epilepsy and their association with cancer varies [3]. As affected patients usually respond to immunosuppressive treatment, they are an important differential diagnosis for psychiatric diseases, cognitive decline, and viral encephalitis. One antibody that does not fit entirely into this classification is anti-Tr. The antigen was initially described intracellular. However, recent work identified the antigen as delta/notch-like epidermal growth factor-related receptor (DNER) a protein also present in the neuronal membrane [4]. 

## Which tests are available – Implementation in the diagnostic laboratory 

Different techniques are available for the diagnosis of anti-neuronal antibodies, each with its value and potential limitations: tissue based assays, immunoblots, cell based assays, ELISA, and immunoprecipitation. For some neuronal/glial antigens, a systematic comparison of different assays has been performed [5, 6]. 

### Tissue-based assays (TBA)

**Principle**

Antibodies that are present in CSF or serum of patients are identified on brain tissue of rodents or primates, using indirect immunofluorescence or immunohistochemistry. 

**Application**

This test is recommended as screening method for Group 1 and 2 antibodies, with the exception of anti-GlyR-antibodies, as they may be not detected in the screening TBA. 

**Implementation**

**Figure 1:**
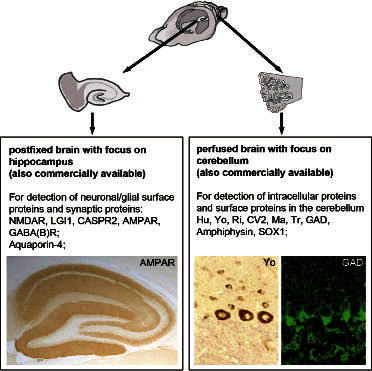
Figure 1. Screening for Group 1 and 2 antibodies with TBA.

Rat brain is obtained and dissected after killing the animal with CO_2_. Two different pretreatment methods are necessary to detect either Group 1 antibodies in the rat cerebellum or Group 2 antibodies in the rat hippocampus (Figure 1) [7]. This approach requires having an animal facility and approval of the ethical committee for the procedure. 

Alternatively, commercially available kits of rodent or primate brain sections can be used. They have the advantage of not requiring animal facilities. However, they are rather expensive. Two different kits have to be purchased for screening either Group 1 or Group 2 antibodies. 

### Immunoblot

**Principle**

Antibodies that are present in CSF or serum of patients will recognize recombinant antigens as specific band, that were transblotted onto a nitrocellulose membrane. 

**Application**

This test is recommended as confirmatory test for Group 1 antibodies with the exception of anti-Tr-antibodies. 

**Implementation**

Different companies provide commercially available immunoblots for the most common onconeuronal antibodies (Hu, Yo, Ri, CV2, Ma1/2, Amphiphysin, SOX1, and GAD) that can easily be implemented in the diagnostic laboratory. Immunoblots from different manufacturers have some variability in sensitivity and specificity that have to be taken into account before deciding for a specific company [5]. Spezialized laboratories provide in-house immunoblots for detection of rare antibodies either with purified proteins or electrophoretically separated extracts of rat cerebellum [8]. 

### Cell-based assays (CBA)

**Principle**

Antibodies that are present in CSF or serum of patients are identified on suitable cell lines (such as HEK293 cells) that are transfected with an eukaryotic expression vector (plasmid) encoding the antigen. 

**Application**

This test is recommended as confirmatory test for Group 2 antibodies and anti-Tr-antibodies. 

**Implementation**

Different companies provide either commercially available plasmids or already transfected cells for the most common surface proteins (NMDAR, LGI1, CASPR2, AMPAR, GABA_B_R,) that can easily be implemented in the diagnostic laboratory. Spezialized laboratories provide in-house cell-based assays for detection of additional antibodies such as GlyR or mGluR1/5. 

### ELISA

Principle: Antibodies that are present in CSF or serum of patients are identified, using an automatic reader that quantifies the reactivity of an enzyme that is activated when there is antibody binding to a specific substrate. 

Application: This test only plays a minor role in the routine diagnostic work-up of anti-neuronal antibodies and is mainly reserved for special issues such as determination of antibody titer. 

### Immunoprecipitation

**Principle**

Antibodies that are present in CSF or serum of patients bind to a specific antigen, the antigen-antibody complex is precipitated out of solution and measured. Since large amounts of substrate are often necessary, serum is preferably used over CSF. 

**Application**

This test is used for the detection of a few specific antibodies, such as voltage-gated calcium channel antibodies and is provided by specialized immunological laboratories. 

## How to test – Algorithmic approach and potential limitations 

To provide a maximum of sensitivity and specificity a combination of a screening method (TBA) and a confirmatory test (immunoblot and CBA) using serum and CSF is recommended [9]. TBA provide an excellent screening tool because they detect the full range of already characterized antibodies and can reveal new antibodies. However, knowledge of reactivity pattern and proper quality managment is essential in maintaining the highest standards in the practice of this diagnostic testing. 

**Figure 2:**
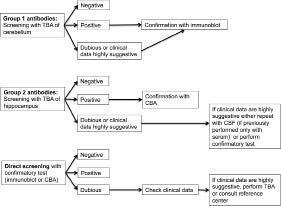
Figure 2. Algorithmic approach to diagnosis of anti-neuronal antibodies.

Commercial confirmatory assays are highly sensitive and specific. Robust signals are diagnostic. However the high sensitivity bears the risk of weak results. Screening with immunoblot occasionally shows very weak or multiple bands, that can be false positive or not significant. Problems related to non-specific background signal also occur with CBA, particularly when only serum is used. If a center decides to perform only confirmatory tests (without preceding screening method), results have therefore be interpreted with caution and put in context with the clinical data. An algorithmic approach to diagnosis of anti-neuronal antibodies is shown in Figure 2. 

## Who should perform testing 

Neurological syndromes associated with anti-neuronal antibodies are rare diseases and diagnosis should be performed in experienced centers to guarantee high diagnostic quality, and to gain a maximum of information for requesting clinicans and people doing research. Smaller departments that decide to offer testing should be aware of diagnostic pitfalls and in case of any doubt consult a reference center. 

## Conclusion 

Accurate detection of anti-neuronal antibodies has important implications for the clinical work-up and treatment of patients with paraneoplastic and autoimmune neurological disorders. Several tissue- and cell-based assays (in-house or commercial) are available to enable early diagnosis. The indicated algorithmic approach provides a maximum of sensitivity and specificity, however, interpretation of results needs experience and should be put in the context of clinical information. Each center should be aware of the value and potential limitations of their individual testing system and in case of doubt consider to consult a reference center. 

## Acknowledgments 

The work was supported in part by the National Institutes of Health R01NS077851, R01MH094741, the National Cancer Institute R01CA089054, Fondo de Investigaciones Sanitarias (FIS, 11/01780), and Fundació la Marató de TV3 (Josep Dalmau), grant FIS PS09/0193, Madrid, Spain (Francesc Graus). RH was funded by the Fonds zur Förderung der wissenschaftlichen Forschung, Austria, Project J3230. 

Dr. Dalmau has a research grant from Euroimmun, and receives royalties from patents for the use of Ma2 and NMDAR as autoantibody tests. Drs. Höftberger and Graus declare no conflict of interest. 

**Table 1:** Classification of anti-neuronal antibodies.

Antigen	Associated syndromes	How to test	Commercially available
Group I			
Hu (ANNA1)	Encephalomyelitis, PCD, LE, brainstem encephalitis, sensory neuropathy	TBA, IB	yes
CV2 (CRMP5)	Encephalomyelitis, Chorea, PCD, LE, sensomotoric neuropathy	TBA, IB	yes
Amphiphysin	SPS, myelopathy and myoclonus, Encephalomyelitis	TBA, IB	yes
Yo (PCA1)	PCD	TBA, IB	yes
Ri (ANNA2)	Brainstem encephalitis, Opsoclonus myoclonus	TBA, IB	yes
MA-2	LE, brainstem encephalitis	TBA, IB	yes
SOX1 (AGNA)	Encephalomyelitis, PCD	TBA, IB	yes
GAD65	SPS, cerebellar ataxia, LE	TBA, IB	yes
Tr (DNER)	PCD	TBA, CBA	no
Group II			
NMDAR	encephalitis	TBA, CBA	yes
LGI1	LE	TBA, CBA	yes
GABA_B_R	LE	TBA, CBA	yes
AMPAR	LE	TBA, CBA	yes
CASPR2	Morvan’s syndrome	TBA, CBA	yes
GlyR	PERM	CBA	no
mGluR1	Cerebellar ataxia	TBA, CBA	no
mGluR5	LE	TBA, CBA	no
VGCC	LEMS, PCD	RIA	yes
Aquaporin-4 (glial)	NMO	TBA, CBA	yes

ANNA = anti-neuronal nuclear antibody; CRMP = collapsin response mediator protein; PCA = purkinje cell autoantibody; AGNA = anti-glial nuclear antibody; GAD65 = glutamic acid decarboxylase 65; DNER = delta/notch-like epidermal growth factor-related receptor; NMDAR = N-methyl-D-aspartate receptor; LGI1 = leucine-rich glioma-inactivated 1; GABABR = gamma-aminobutyric acid-B receptor; AMPAR = amino-3-hydroxy-5-hydroxy-5-methyl-4-isoxazolepropionic acid receptor; CASPR2 = contactin-associated proteinlike 2; GlyR = glycine receptor; mGluR1/5 = metabotropic glutamate receptor type 1/5; VGCC = voltage-gated calcium channel; PCD = paraneoplastic cerebellar degeneration; LE = limbic encephalitis; SPS = stiff-person syndrome; PERM = progressive encephalomyelitis with rigidity and myoclonus; LEMS = Lambert-Eaton myasthenic syndrome; NMO = neuromyelitis optica.
